# Simulation of Reflections from the Underlying Surface in an On-Board Radar with SAR

**DOI:** 10.3390/s26092742

**Published:** 2026-04-28

**Authors:** Vladimir Yu. Volkov, Vadim A. Nenashev

**Affiliations:** 1 Department of Radioengineering, Saint-Petersburg State Electrotechnical University (LETI), 197376 Saint Petersburg, Russia; 2 Department of Design and Technology of Electronic and Laser Devices, Saint-Petersburg State University of Aerospace Instrumentation (SUAI), 190000 Saint Petersburg, Russia; nenashev.va@gmail.com

**Keywords:** speckle images, rough reflective surface, probability density function, K-distribution, generalized Gaussian distribution, cumulants

## Abstract

This study investigates the selection of suitable statistical models for speckle reflections from the underlying surface under low-altitude sensing conditions. A parametric approach to modeling speckle images of terrain fragments typical of synthetic aperture radar (SAR) is presented. We use a phenomenological model of speckle formation during radio wave interference, taking into account the spectrum of fluctuations, the roughness of the reflecting surface, the angle of incidence, and other radar parameters. We investigate the influence of the properties of the reflecting surface and the probing parameters on the nature of speckle images. The values of the sample cumulative coefficients for various multiplicative models of the reflection distribution are obtained. The properties and characteristics of various classes of distributions for describing the intensity and amplitude of speckles are considered: the gamma distribution, the K-distribution, and the classes of non-Gaussian probability densities G and G^0^. A generalized Gaussian (GG) distribution is used to model the complex components of reflected signals. We compare the obtained model characteristics with the sample characteristics of real terrain fragments in synthesized speckle images obtained by the on-board radar system. Based on a comparative analysis of cumulants, this paper examines methods for modeling amplitude and intensity speckle images using several classes of backscatter probability densities. Limitations in specific applications have been identified, and a modeling method using quadrature components has been developed in cases of extremely rough reflections.

## 1. Introduction

Modeling synthesized radar images is essential for detecting and localizing objects of interest, as well as for analyzing the properties of the underlying surface in radar monitoring applications. The properties of synthesized radar images depend on the type and properties of reflections, on the type of irradiated signal and antenna parameters, as well as on the geometric sensing scheme [1]. The roughness and geometry of the surface, its humidity, and electrical properties determine the depth of radiation penetration and the nature of its scattering. The wavelength of the radiation, the polarization, the type of probing signal, and the probing scheme affect the formation of the radar image, which describes both the amplitude and phase pattern of reflections.

Radar images acquired by spaceborne, airborne, helicopter-borne, and unmanned platforms exhibit distinctive features. However, the presence of speckle structures (spots) is common in amplitude images, due to the coherent interaction of reflected waves [1,2,3]. On-board surveillance systems always have reflections from the underlying surface, which is modeled by a random homogeneous spatial field describing the heights of the roughness. The initial parameter for modeling such a field is the width of the spatial spectrum of fluctuations, which is related to the length of the spatial correlation between two points on the surface.

In addition to the properties of the rough surface, the radar speckle image is influenced by the way it is formed, taking into account the geometric sensing scheme. The main factors here are the looking angle, usually measured from the vertical, the height and speed of the carrier, the range of radiated frequencies, and the type of signal modulation [4,5,6,7].

Some important results in this area have been obtained in recent years, but “it is obvious that it is impossible to create a universal SAR simulator, no matter how desirable it may be, since it must be specially adapted for its intended use. On the other hand, it is possible to create a simple SAR simulator framework that is common to many different applications” [5]. At large looking angles, effects such as distortion of geometric relationships and shading of some objects by others occur [8]. These effects require separate consideration.

Objects of interest often appear in images as areas of heterogeneity or as borders. Their modeling is a separate task [6]. To identify and recognize objects of interest in on-board systems, it is useful to have a set of parametric speckle image models for frequently occurring fragments of the underlying surface, such as woodlands, fields, arable land, water surfaces, etc. Despite the variety of known parametric models for reflections, there is currently no clear understanding of the applicability of a particular model, as well as their inherent limitations.

This paper addresses this problem by analyzing the applicability of several parametric models to SAR speckle images of the underlying surface. The use of a well-known phenomenological model of phase fluctuations caused by a rough reflecting surface when probing with plane-polarized radiation at a certain angle of inclination is considered. Based on the multiplicative model, we analyze the influence of various statistics of fluctuations of the reflecting surface on the cumulative characteristics of speckle images. The speckle images obtained by the simulation are compared with the real speckle images of the underlying surface obtained by on-board systems in the S wave band and X wave band.

## 2. Previous Work

Efforts to create a universal model of reflected radar signals have been made over the past 50 years. In many ways, they relied on studies of the interaction of optical signals [2]. One of the first reviews of models applied to radar signals is given in [3]. The greatest success has been achieved in the development of parametric statistical models that rely on the central limit theorem and the hypothesis of a set of homogeneous reflectors.

The standard approach to SAR image modeling defines the reflected signal as a complex one, where the real and imaginary components satisfy the following assumptions [9]: (1) the number of scatterers is large, (2) the scatterers are statistically independent, (3) the instantaneous scattering phases are statistically independent of the amplitudes, (4) the phase is uniformly distributed, (5) the reflectors are relatively small when compared to the illuminated scene, and (6) there is no dominating scatterer in the scene.

For a homogeneous reflecting surface in the single-look case, the reflected signal power is classically modeled by an exponential distribution. For multiple independent observations (multi-look mode), this model leads to a gamma distribution *v* ~ Г(*L*, β), where *v* = *u*^2^, and *u* is the amplitude of the signal. The effective number of independent looks *L* is a parameter of the shape of the distribution, and β affects the power of the reflected signal [10]. This description is in good agreement with the simulation results based on the phenomenological model. However, the limitations of the model do not allow us to approach the real characteristics of speckles, the distributions of which often have significant differences, for example, heavier distribution “tails”. Estimates of real probability densities give higher values of the coefficient of skewness and kurtosis than for the gamma distribution. A fairly complete overview of one-dimensional statistical models for describing reflections from the sea surface is given in [11]. Attempts to take into account the correlation properties of reflected signals for modeling purposes were made in [12,13,14,15].

More advanced approaches employ multiplicative models that account for fluctuations in the effective scattering area of the reflecting surface. The gamma model can be extended through compounding to account for fluctuations of the reflecting surface. As a result, a number of probability distributions for the amplitude and intensity of the image were proposed, such as the K-distribution, G-distribution, as well as their constructive generalizations [16,17].

This paper examines several variants of this approach and their inherent limitations for speckle modeling. However, this approach did not allow us to achieve an acceptable description of the radar speckle image. In particular, it was not possible to model statistics with low coefficients of variation and negative values of skewness and kurtosis. The main limitation seems to be the Gaussian distribution for the quadrature components of the radar signal. In fact, these components may have substantially non-Gaussian distributions. Apparently, Frery A. C. was one of the first to describe the statistical properties of these components using non-Gaussian models [18]. One practical option for modeling quadrature components is to use a generalized Gaussian (GG) distribution. Another option is an alpha-stable distribution [9,19].

Existing speckle models for on-board surveillance systems are often weakly linked to the actual sensing scenario and do not adequately account for system parameters (radiated frequencies and types of signal modulation) and registration schemes (heights, sensing angles, and flight speeds). The relationship of image models with some of these parameters was investigated in [20] for static registration schemes. There are practically no research results for models of synthesized radar images obtained for dynamic registration schemes during the movement of a low-flying airborne carrier. These issues require further careful consideration. A comparative cumulant-based analysis of existing parametric models reveals their applicability limits in specific sensing scenarios and motivates a modeling approach based on quadrature components for extremely rough reflections. One of the non-Gaussian distributions for quadrature components is also modeled in order to more adequately describe reflections from various fragments of a rough surface, taking into account its correlation and the sensing angle.

## 3. Modeling Reflections from a Rough Surface

### 3.1. Formation of a Rough Surface

**Figure 1:**
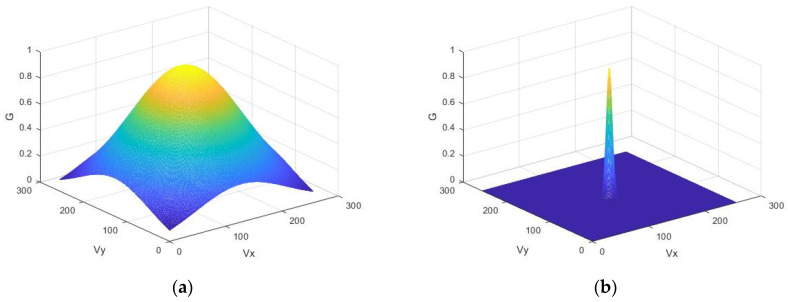
Normalized power spectrum for rough surface: (**a**) for spectral width *B* = 2; (**b**) for *B* = 0.1.

Let the radar irradiate a rough surface at a certain angle θ to the normal. The heights of the rough reflecting surface are described by a random two-dimensional function *h*(*x*,*y*). It is defined on a discrete grid and modeled by a random field with a given correlation function. A widely used phenomenological model based on random phase relationships [21] is adopted to describe coherent reflection from a rough surface. The heights of the rough reflecting surface are described by a random two-dimensional function *h*(*x*,*y*). It is defined on a discrete grid and modeled by a random field with a given energy spectrum *G*(ν*_x_*,ν*_y_*), which is assumed as Gaussian with width *B*; v_x_ = 2π/*dx* and v_y_ = 2π/*dy* are spatial frequencies in rad/m. Figure 1 shows two normalized power spectra for different values of parameter *B*, which determines the width of the spectrum. The values *dx* and *dy* are normalized to the wavelength λ so that the width of the spectrum *B* is expressed in fractions of the wavenumber *k*_0_ = 2π/λ.

Roughness is the reason for the appearance of a speckle structure under coherent irradiation. The phase of each spectral component of the reflections is represented by an independent and uniformly distributed random variable. The degree of surface roughness measured in wavelengths is determined by the *RU* parameter, which regulates the range of random phase changes. This parameter determines the proportion of random phase changes in the spectrum, which are maximal at *RU* = 1. A smooth surface is obtained at *RU* = 0. The spectrum of the rough surface, taking into account amplitude fluctuations and the two-way propagation, is expressed by the formula [22,23]

$$g(v_{x},v_{y})=\sqrt{G(\nu_{x},\nu_{y})}\exp(j4\pi RU\cdot \mathrm{rand}(\nu_{x},\nu_{y})),$$

where rand(*ν_x_*,*ν_y_*) gives a random value with a uniform distribution in [0, 1].

**Figure 2:**
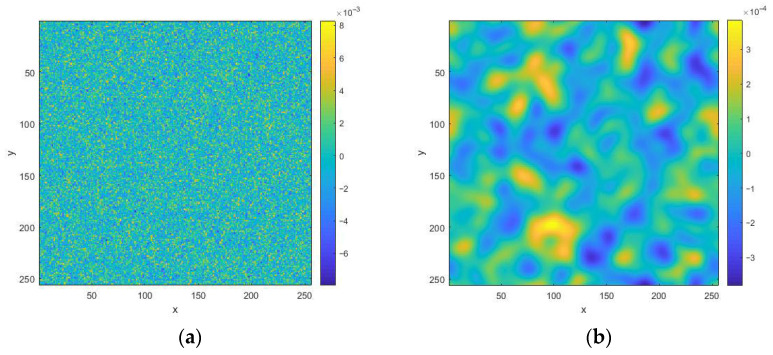
Normalized height function images *h*(*x*,*y*) for rough surface with *RU* = 1: (**a**) for spectral width *B* = 2; (**b**) for *B* = 0.1. The distances normalized to the radiation wavelength are plotted along the axes.

To form random realizations of rough surface heights, it is necessary to take the inverse Fourier transform of the spectral function *g*(ν*_x_*,ν*_y_*). The latter is a two-dimensional matrix that is not Hermitian. As a result, the inverse two-dimensional Fourier transform leads to a loss of symmetry properties for the field of random heights, and it becomes inhomogeneous. To correct this situation, a Hermitian conjugate spectral function *g*^H^(ν*_x_*,ν*_y_*) should be formed, taking into account the cyclicity of the arguments. Then, the spectrum $gh(v_{x},v_{y})=(g(v_{x},v_{y})+g^{H}(v_{x},v_{y}))/2$ becomes Hermitian, and the inverse Fourier transform gives a real function of the heights of the surface $h(x,y)=FFT\{gh(v_{x},v_{y})\})$. The images of the normalized height functions are shown in Figure 2 for two values of the spectrum width.

When forming the reflecting surface, two significant assumptions are used: the Gaussian shape of the fluctuation power spectrum and the uniform phase distribution of the reflected signals. As a result, a Gaussian model of the reflecting surface is obtained, which leads to a Gaussian distribution for the quadrature components of the reflected signal and already at this stage significantly limits further application for modeling real reflections. This limitation can be partially removed by the subsequent nonlinear transformation of the quadrature components during modeling, which will be discussed further.

### 3.2. Modeling of Reflectivity

When vertically probing the surface, the height values are converted into random changes in the range to the surface areas. The latter are involved in the formation of random phase shifts of signals $\mathrm{phase}(x,y)=2k_{0}h(x,y)$. When irradiating a rough surface at a certain angle θ (look angle) to the normal, the phase shifts are calculated taking into account the surface gradients *hx* and *hy*. The correction $2k_{0}(hx(x,y)\sin(\theta )+hy(x,y)\sin(\theta ))$ is subtracted from the previous phase value, taking into account the looking angle:

$$\mathrm{phase}(x,y)=2k_{0}(h(x,y)\cos(\theta )-(hx(x,y)\sin(\theta )+hy(x,y)\sin(\theta )).$$

**Figure 3:**
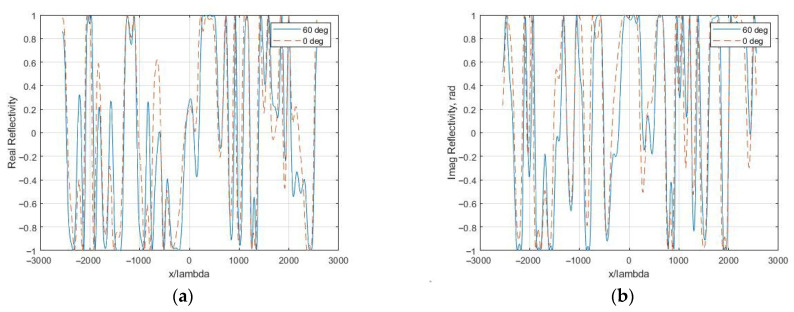
Normalized reflectivity during vertical and inclined probing of a rough surface at different sliding angles θ, *RU* = 1, and *B* = 0.1: (**a**) real part; (**b**) imaginary part.

Surface reflectance is $R(x,y)=R_{0}(x,y)\cdot \exp(j\cdot \mathrm{phase})$, where $R_{0}(x,y)$ describes the amplitude changes, for example, due to terrain slopes, shadowing, as well as the amplitude pattern of the antenna. The influence of these factors, as well as the penetration of radiation under the surface, on signal statistics requires separate consideration [24]. In this work, it was assumed that this was true for local surface areas. Figure 3 shows the changes in reflectivity during vertical (θ = 0°) and inclined probing (θ = 60°) due to phase differences. The distance normalized to the wavelength is plotted along the abscissa axis, and the real and imaginary components of reflectivity are plotted along the ordinate axis.

The figures show that the looking angle significantly affects the phase of the complex reflectivity and*,* consequently, the statistical characteristics of speckle images.

### 3.3. Speckle Image Modeling in an On-Board Radar with SAR

The case of a side-looking radar system at an angle of θ = 60° during horizontal flight of the carrier with a constant velocity of *V* = 50 m/s at an altitude of *H* = 35 m is chosen for analysis. With a synthesized aperture length of *L_SAR_* = 100 m, the synthesis time is *T*_SAR_ = 2 s. Probing with pulses lasting 2 ns provides a range resolution of 0.3 m. At a wavelength of 0.1 m and a pulse repetition rate of Fr = 240 Hz, 480 pulses are involved in synthesis.

Processing of reflected signals on board includes two stages: obtaining a “raw” image and a synthesis stage implemented by correlation processing [4,5,6,7]. At the stage of forming the “raw” image, the amplitude and phase of the reflectivity are read for each range and angle discretion using the value of the current inclined range, which takes into account the movement of the carrier and noise. Next, the resulting image is “compressed” by range and angle through correlation processing. The reference signals are calculated according to the specified parameters of the geometric scheme.

**Figure 4:**
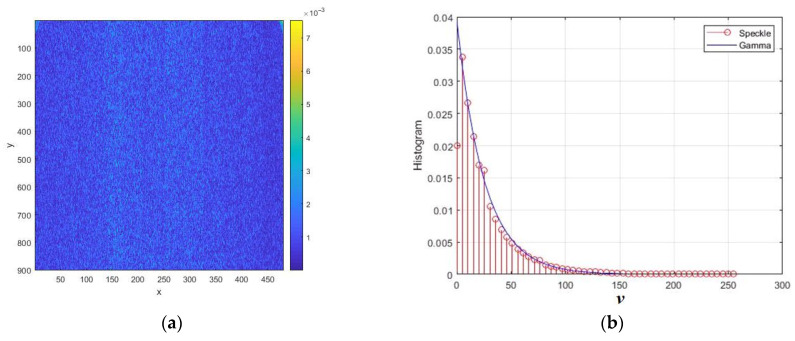
Simulation results of intensity speckle image modeling for rough surface at *RU* = 1, *B* = 0.1, and θ = 60°: (**a**) speckle intensity image; (**b**) normalized intensity histogram. The distances normalized to the radiation wavelength are plotted along the axes *x* and *y*.

The normalized intensity speckle image obtained by modeling reflections from a surface with roughness is shown in Figure 4a. Figure 4b shows the normalized intensity speckle histogram and its approximation by an exponential distribution with an equivalent scale parameter. The intensity speckle histogram looks like an exponential relationship. This model is often used because it has a physical justification for single looking [2,10]. However, in the case of SAR, the analysis of the cumulative sample values reveals significant differences from the exponential model. In particular, the third cumulative coefficient turned out to be 0.86 instead of 2, and the fourth was 1.21 instead of 6.

**Figure 5:**
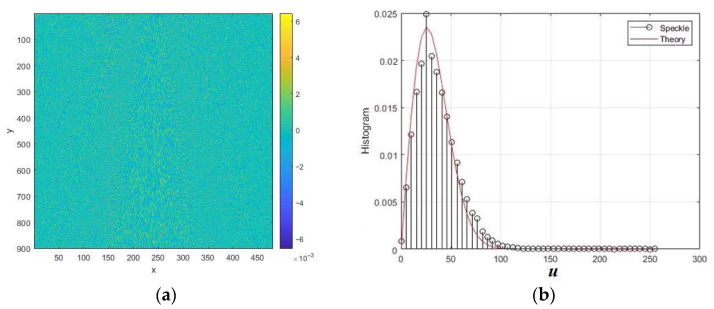
Simulation results of the amplitude speckle image for a rough surface with *RU* = 1, *B* = 0.1, and θ = 60°: (**a**) the image of the amplitude speckle; (**b**) the normalized histogram of the amplitude. The distances normalized to the radiation wavelength are plotted along the axes *x* and *y*.

The normalized amplitude speckle is shown in Figure 5a, and in theory it should have a Rayleigh distribution density. However, the differences between this density and the histogram are already more noticeable. These differences are even more evident in the values of the cumulative coefficients.

The classical model is the gamma speckle intensity distribution, which is used for repeated looks [2,3,10,11]. However, it can be seen that even with the above assumptions related to the Gaussian spectrum of the surface and the uniform phase distribution, the cumulant characteristics of the speckle images obtained using SAR synthesis modeling do not correspond to the characteristics of classical distributions. This conclusion is all the more obvious for quadrature images.

**Figure 6:**
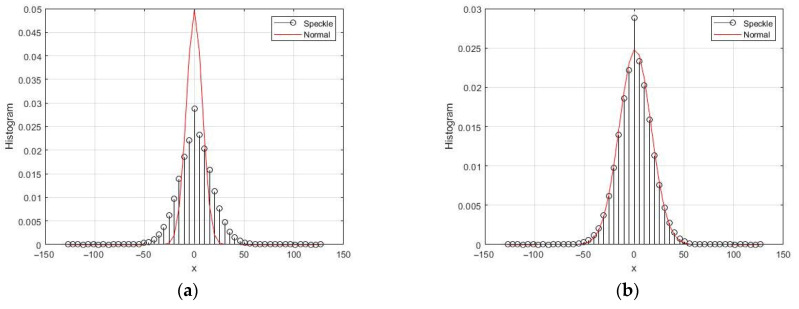
Histograms of the normalized quadrature component for speckle from the rough surface at *RU* = 1, *B* = 0.1, and θ = 60°: (**a**) in comparison with normal density; (**b**) in comparison with normal density with increased variance.

The histogram of the normalized quadrature component of the speckle (Figure 6a) differs from the theoretical Gaussian curve with equivalent parameters. Differences from the normal law are also confirmed by estimates of the kurtosis coefficient, which in this case is 0.35, although it is 0 for the Gaussian distribution. Thus, the results of speckle image modeling in this case raise doubts about the validity of the classical gamma distribution model for intensity and Rayleigh density for amplitude in the case of a single look.

The question of the applicability of the Gaussian distribution to describe the distribution of quadrature components is of interest. In Figure 6b, the Gaussian density is used for approximation, but its variance is increased by 4 times. The attractiveness of Gaussian approximation lies in the possibility of obtaining the moment and correlation characteristics of speckles by calculation. This allows us to create constructive parametric models to describe various fragments of the underlying surface in order to recognize them, as well as enrich the samples for training the neural network.

The gamma intensity distribution model is based on the Gaussian distribution of quadrature components. Various promising models are built on this approximation, in particular, a family of multiplicative models, which are investigated further. In this case, the results of speckle image modeling raise doubts about the validity of the classical model of the gamma distribution for intensity, the Rayleigh density for amplitude, and the Gaussian distribution for quadrature components in the case of a single SAR survey.

The following sections explore the limitations of known models based on Gaussian distributions for quadrature components and the applicability of the gamma speckle intensity distribution and the corresponding chi speckle amplitude distribution to describe synthesized images in an on-board radar with SAR.

## 4. Modeling of Speckle Images with Gamma and Chi Distributions

The gamma distribution is a common model for describing the intensity of speckles upon repeated viewing. The speckle image is formed by summing the speckle images obtained for a single look (*L* = 1). The probability density of the gamma distribution $f(v)={\left(1/\beta \right)}^{L}v^{L-1}\exp(-v/\beta )/\Gamma (L)$, where Г(x) is the gamma function, is denoted as v~Γ(L,β). Using mathematical expectation μ = β*L*, we can obtain the form given in [9]: $f(v)={\left(L/\mu \right)}^{L}v^{L-1}\exp(-vL/\mu )/\Gamma (L)$.

The cumulants are equal to $\kappa_{n}=(n-1)!L\beta^{n}$. When the scale parameter value β = 1/*L*, the density takes the form [18] $f(v)=L^{L}v^{L-1}\exp(-vL)/\Gamma (L)$, and the mathematical expectation is one for any value of *L*. The shape parameter *L* of the gamma distribution is related to the effective number of independent radar looks [10] and may be fractional.

For amplitude speckles, a nonlinear transformation (the square root of the gamma distribution) yields the chi distribution $f(u)=u^{k-1}\exp(-u^{2}/2\beta^{2})/(2^{k/2-1}\beta^{k}\Gamma (k/2))$, which is often denoted as $u\sim \Gamma^{0.5}(k,\beta )$ [9], where *k* = 2*L* is the number of degrees of freedom. The formula $m_{n}=2^{n/2}\beta^{n}\Gamma ((k+n)/2)/\Gamma (k/2)$ for the moments of this density allows us to calculate the theoretical values of the cumulative coefficients.

**Figure 7:**
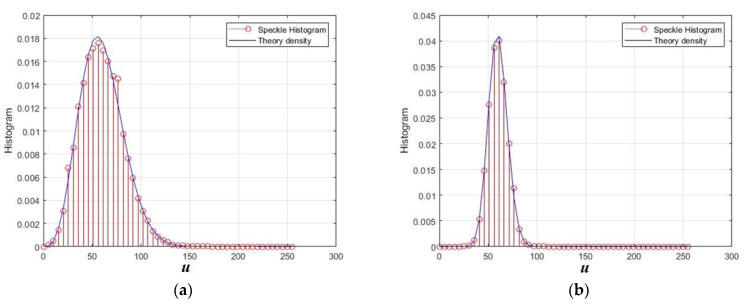
Chi distribution densities with corresponding histograms: (**a**) *L* = 2; (**b**) *L* = 10.

Two examples of normalized chi distribution densities and histograms obtained by simulation are shown in Figure 7 for *L* = 2 and *L* = 10. Gaussian models of quadrature components were used in the formation of histograms. As the number *L* of independent observations increases, the densities narrow.

**Figure 8:**
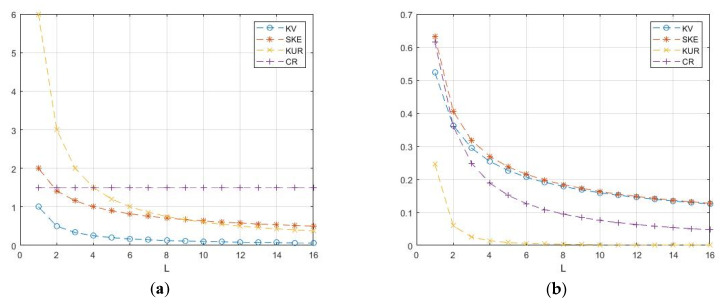
Theoretical dependences of cumulative characteristics: (**a**) gamma distribution; (**b**) chi distribution.

The analysis of these models shows a rather restrictive cumulative composition for describing real situations. Consider four characteristics: KV=σ/m—coefficient of variation; $\mathrm{SKE}=\kappa_{3}/\sigma^{3}$—coefficient of skewness; $\mathrm{KUR}=\kappa_{4}/\sigma^{4}$—coefficient of kurtosis; and $\mathrm{CR}=\mathrm{KUR}/{\mathrm{SKE}}^{2}$—cumulative ratio. Calculations of these characteristics for the selected models (Figure 8) show their relatively small values, which also decrease with increasing *L* [20]. For speckle amplitudes, these characteristics have lower values than for intensities, so amplitudes have less fluctuations than intensities.

The model under consideration does not allow for obtaining large values of the coefficient of variation, as well as large and negative values of asymmetry and kurtosis, which is the case in real situations.

## 5. Perspective Models of Speckle Images

The characteristics of gamma speckles and chi speckles turn out to be different from the actual characteristics of reflections in airborne radars [9,16,22,23]. To improve the model, complication (compounding) of the initial density for intensity or amplitude is applied. It is considered conditional for a fixed value of the scale parameter. This parameter is related to the power of the reflected signal, i.e., the area of the effective scattering surface (radar cross-section). Next, this scale parameter is assumed to be random, and its own distribution is introduced for it, which has its own scale and shape parameters that are added to the parameters of the unconditional density. Changes in the entered parameters expand the capabilities of the resulting model, since they allow us to take into account fluctuations in the area of the reflecting surface. Usually, the multiplicative model *z* = *ux* is used for modeling, where *u* is the initial speckle pattern, and *x* is the fluctuations of the reflecting surface [16,17].

The gamma distribution for the average reflection intensity *x* ~ Г(*a*,*b*) is physically justified and, together with the gamma speckle intensity *u ~* Г(*L*,1/*L*), leads to a model of the K_I_-speckle intensity distribution. For the speckle amplitudes, the chi distribution of the multiplicative components *x* ~ Г^0.5^(*a*,*b*) and *u* ~ Г^0.5^(*L*,1/*L*) are used, and these lead to the K_A_-distribution model of amplitude reflections.

Other compounding options do not have such a clear physical justification. In particular, other authors [18,22,23] proposed two density classes to describe the amplitude reflections in the chi distribution of the initial speckle *u* ~ Г^0.5^(*L*,1/*L*):

- Class G_A_, for which the density of fluctuations is described by the square root of the generalized inverse normal distribution *x ~* N^–0.5^(α,γ,λ), with G_A_-density
  $$f(x|\alpha ,\gamma ,\lambda )={\left(\lambda /\gamma \right)}^{\alpha /2}x^{2\alpha -1}\exp(-\gamma /x^{2}-\lambda x^{2})/K_{\alpha }(2\sqrt{\lambda \gamma }),$$
  where *K*_α_(∙) is a modified Bessel function of the second kind;
- Class G^0^_A_, for which the density of fluctuations is represented by the inverse chi distribution *x* ~ Г^−0.5^(α,γ) (instead of the direct Г^0.5^(α,γ), which resulted in a K_A_-distribution)

$$f(x|\alpha ,\gamma )=2x^{2\alpha -1}\exp(-\gamma /x^{2})/\gamma^{\alpha }\Gamma (-\alpha ).$$

Here, the α parameter is negative. Such a distribution can be obtained by inversely converting *x* = 1/*y* random values with a chi distribution *y* ~ Г^0.5^(*a*,*b*), with α = −*a* and γ = 1/*b*.

Then, for the amplitude speckle *z* = *ux*, we get a distribution in the G^0^_A_ class

$$f_{A}(z|L,\alpha ,\gamma )=2L^{L}\Gamma (L-\alpha )z^{2L-1}/\gamma^{\alpha }\Gamma (L)\Gamma (-\alpha ){\left(\gamma +Lz^{2}\right)}^{L-\alpha }.$$

It was shown [18] that for a homogeneous surface, the G_A_ class corresponds to the K–distribution, and in the case of an extremely inhomogeneous surface, it passes into the G^0^_A_ class.

For the intensity speckle pattern, we assume a gamma distribution for the speckle component u ~ Г(L,1/L) with inverted gamma distribution x ~ Г–1(α,γ) for fluctuations. Finally, it leads to the G^0^_I_ class:

$$f_{I}(z|L,\alpha ,\gamma )=L^{L}\Gamma (L-\alpha )z^{L-1}/\gamma^{\alpha }\Gamma (L)\Gamma (-\alpha ){\left(\gamma +Lz\right)}^{L-\alpha }.$$

### 5.1. K-Distributed Speckle Images

**Figure 9:**
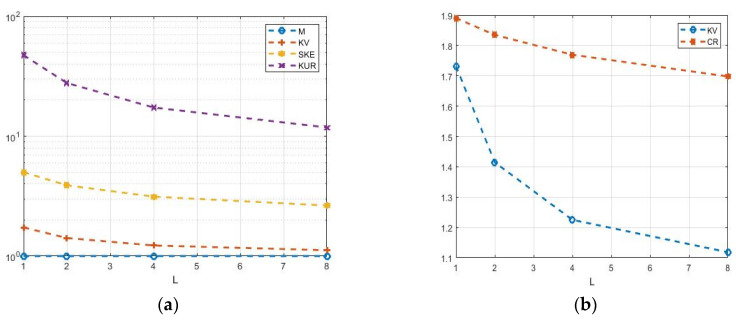
Parameters of the K-distribution as functions on degrees of freedom *L*: (**a**) M—expectation, KV—coefficient of variation, SKE—skewness, and KUR—kurtosis; (**b**) CR—cumulative ratio.

The cumulative characteristics of the K-distribution are shown in Figure 9 as a function of *L* [13,16]. The coefficients of variation of KV vary from 1 to 1.73, which is slightly higher than that of the gamma density. The cumulative CR coefficient varies from 1.5 to 1.9, which may still be insufficient.

The speckle model with a K-distribution allows us to obtain a wider range of characteristics than the model with a gamma distribution. However, negative skewness and kurtosis values and small coefficients of variation are not achieved in principle, although such values are found in real speckle images.

### 5.2. Intensity Speckle in the Class G^0^_I_

Suppose a speckle has gamma intensity distribution *u* ~ Г(*L*,1/*L*) and reflections obey inverted gamma density *x* ~ Г^–1^(α,γ) [23,25,26,27,28]:

$$f(x|\alpha ,\gamma )=\gamma^{-\alpha }x^{\alpha -1}\exp(-\gamma /x)/\Gamma (-\alpha ).$$

**Figure 10:**
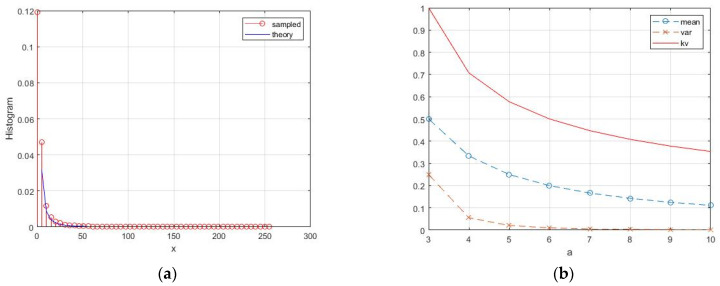
Inverted gamma distribution: (**a**) normalized histogram for *a* = 1; (**b**) theoretical values of the mean, variance, and coefficient of variation.

**Figure 11:**
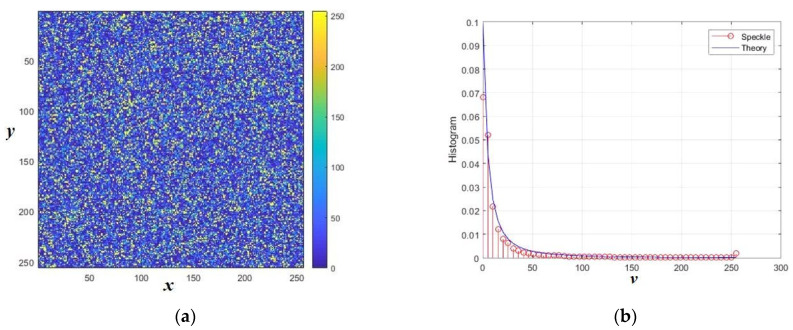
Normalized intensity speckle pattern in the class G^0^_I_ (**a**) and its histogram (**b**). The distances normalized to the radiation wavelength are plotted along the axes *x* and *y*.

A random variable with this distribution can be generated by the inversion *x* = 1/*y* with *y* ~ Г(*a*,*b*) and *b* = 1/γ. The highest values of expectation and variance are realized at *a* = 1, which corresponds to α = −1. This value reflects the degree of heterogeneity of the reflecting surface [23,27,28]. The normalized histogram for this case is shown in Figure 10a. With increasing *a*, the density narrows and its variation decreases. Theoretical values of basic parameters are shown in Figure 10b. Figure 11 shows the speckle pattern and histogram for the noisiest case of extremely heterogeneous clutter: α = −1 for single-look mode *L* = 1.

The probability density and statistical moments (α<−n) for the result of compounding *z* = *xy* are given by the formulas [23,27]:

$$f(z|\alpha ,\gamma )=\frac{L^{L}\Gamma (L-\alpha )}{\gamma^{\alpha }\Gamma (-\alpha )\Gamma (L)}\frac{z^{L-1}}{{\left(\gamma +zL\right)}^{L-\alpha }},\;E(z^{n})={\left(\frac{\gamma }{L}\right)}^{n}\frac{\Gamma (-\alpha -n)\Gamma (L+n)}{\Gamma (-\alpha )\Gamma (L)}.$$

**Figure 12:**
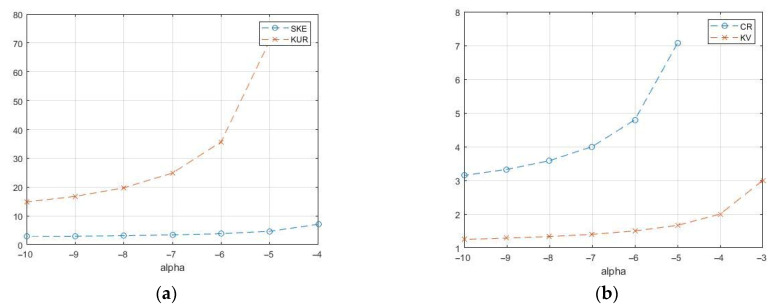
Theoretical values of cumulative coefficients for G^0^_I_ speckle: (**a**) coefficients of skewness and kurtosis; (**b**) cumulative ratio and coefficient of variation.

If we fix the mathematical expectation, then the parameters γ, α, and *L* turn out to be related to each other. The case of the strongest fluctuations corresponds to a single look of *L* = 1, in which case for E(z)=1 we have γ = −α–1. The formulas allow us to calculate the theoretical values of the coefficient of variation and the coefficient of kurtosis, which are shown in Figure 12 depending on α.

**Figure 13:**
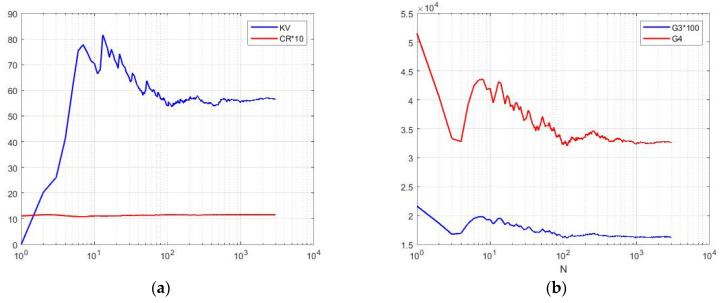
Sample cumulative characteristics of speckle pattern in the class G^0^_I_ depending on sample size *N*: (**a**) KV—coefficient of variation; CR—cumulant ratio (it is increased 10 times on the graphs); (**b**) G3—skewness; G4—kurtosis (it is increased 100 times on the graph).

Compared to previous models, the speckle pattern has significantly higher cumulative coefficients. For the case α = −1 and *L* = 1, the theoretical values of the power moments and cumulative coefficients cannot be calculated, except for mathematical expectation. The sample values of the cumulative coefficients were obtained by modeling and are shown in Figure 13 for the number of *N* samples up to 1000. Speckle distributions can have rather large coefficients of variation (up to several tens), a skewness of the order of 150, and a kurtosis exceeding 30,000. At the same time, the cumulative ratio remains on the order of one, which is quite often observed in real speckle images.

The implementation of the multiplicative model makes it possible to obtain a wide range of speckle characteristics. Distributions from the class G^0^ are indexed by the three parameters [18,22,23,25,26,27,28]. The α parameter is a texture parameter, which is related to the roughness or number of elementary backscatters of the target. Values close to 0 (typically above −3) suggest extremely textured targets, such as urban zones. As the value decreases, it indicates regions with moderate texture (usually α ∈ [−6, −3]), such as forest zones. Texture-less targets, e.g., pastures, usually produce α ∈ (−∞, −6).

The γ parameter is related to the brightness and is called the scale parameter, which influences the signal-to-noise ratio; *L* is the number of looks. However, this model cannot provide the negative values of skewness and kurtosis that occur on real fragments of synthesized images.

### 5.3. Amplitude Speckle in the Class G^0^_A_

**Figure 14:**
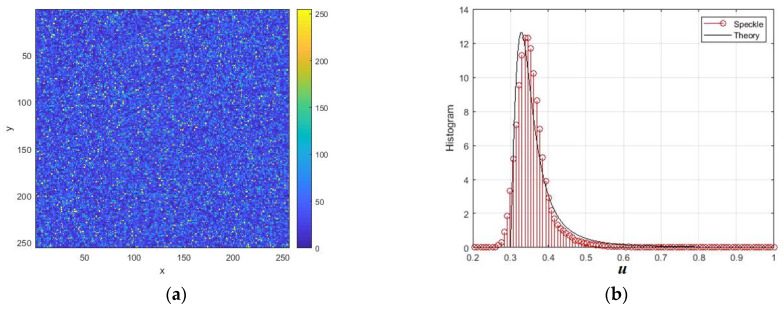
Normalized speckle pattern in the class G^0^_A_ (**a**) and its histogram in comparison with theoretical probability density (**b**). The distances normalized to the radiation wavelength are plotted along the axes *x* and *y*.

The simulation results are shown in Figure 14, where the speckle image (a) and histogram (b) are represented for α = −1 for single-look mode *L* = 1. This is the noisiest case of extremely heterogeneous clutter. It is characterized by significantly heavy distribution tails, which is manifested in cumulative characteristics.

**Figure 15:**
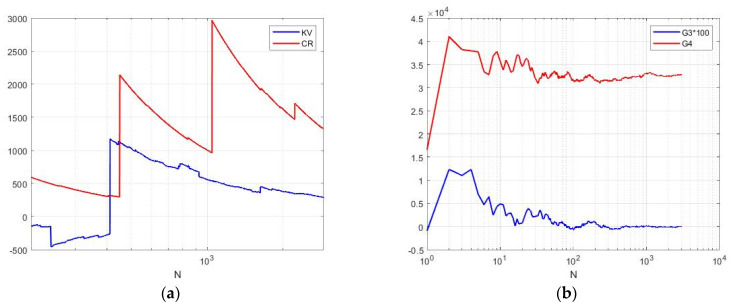
Sample cumulative characteristics of speckle pattern in the class G^0^_A_: (**a**) KV—coefficient of variation; CR—cumulative ratio; (**b**) G3—sampled skewness (the values are increased 100 times on the graph); G4—sampled kurtosis.

Figure 15 shows sampled values of cumulative characteristics, which are calculated for a number *N* of samples till 1000. This single-look case corresponds to the largest sampled cumulative coefficients G3 = 252 and G4 = 64245. In this situation, the coefficient of variation can take on significantly large values, on the order of 2.2, and the cumulative ratio is on the order of 2.5. Such values were unattainable in previous models. Repeated looks, as well as a decrease in the parameter α, which describes amplitude fluctuations, lead to a decrease in the values of these characteristics. Already for *L* = 1 and α = −2 we have G3 = 54; G4 = 5805. At *L* = 8 and α = 10, these indicators decrease to G3 = 1.67; G4 = 5.07, which is quite comparable with the exponential distribution for the classical speckle model.

The sample cumulative characteristics of the amplitude speckle correspond to those obtained for the intensity speckle. In general, the G^0^ model proves to be very useful for modeling a number of fragments of a SAR image of an area. The usefulness of the G*^0^_A_* model for describing extremely heterogeneous land clutter, especially from urban regions, is noted, for example, in [29], where its further development was proposed. However, the model is unable to describe a number of situations in which small coefficients of variation occur with large positive and sometimes negative values of skewness and kurtosis. It seems that the limitations of this model are related to the Gaussian distribution of quadrature components, which are used to form conditional gamma speckle distributions.

### 5.4. Generalized Gaussian Distribution for Quadrature Components

**Figure 16:**
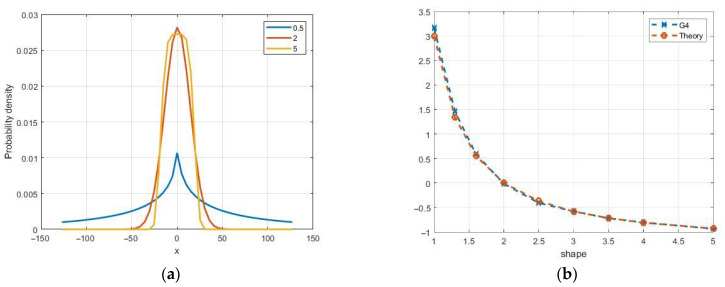
Theoretical probability density functions of the GG distribution (**a**) and kurtosis dependency on the shape parameter γ (**b**) for simulated (G4) and theoretical values.

The considered models for intensity and amplitude speckle used Gaussian distributions for the quadrature components of the synthesized signal. This limited the ability to customize the statistical characteristics of the generated models. The constructive idea is based on the nonlinear transformation of quadrature components in order to gain more control over statistical characteristics. One of the useful models is based on the generalized Gaussian distribution for quadrature components [30]:

$$f(x|\gamma ,\beta ,\mu )\;=\gamma \exp(-{\left|x-\mu \right|}^{\gamma }/\beta^{\gamma })/(2\beta \Gamma (1/\gamma )\;,$$

where γ is the shape parameter, β is the scale, and μ is the location parameter. Theoretical probability density functions of GG distribution are presented in Figure 16a for μ = 0, β = 20, and shape parameter γ = 0.5, 2, and 5. This distribution includes Gaussian and Laplace distributions as special cases for γ = 2 and γ = 1, respectively. The variance of the distribution is $\sigma^{2}=\beta^{2}\Gamma (3/\gamma )/\Gamma (1/\gamma )$. The coefficient of skewness is zero, and the coefficients of kurtosis $\mathrm{KUR}=\Gamma (5/\gamma )\Gamma (1/\gamma )/\Gamma {\left(3/\gamma \right)}^{2}-3$. In particular, for Laplace density, KUR = 3, and for γ > 2, it becomes negative (Figure 16b).

This model may be useful for generating both quadrature components of the speckle image. In Refs. [9,31,32,33], its application for the formation and description of amplitude speckle was considered. Nonlinear transformations are used to obtain non-Gaussian quadrature components in modeling process. Since speckle distributions in quadrature channels are assumed to be Gaussian, the algorithm involves converting statistics to a uniform distribution using the inverse function of the normal distribution. The subsequent transformation is based on the inversion method, taking into account the parameters of the GG distribution. It includes a numerical solution of the corresponding equation for quantiles. The resulting speckles of the quadrature components have significant differences in higher cumulants from the Gaussian distribution, which expands the model’s capabilities to describe various reflections. Figure 16b shows the sample values of the kurtosis coefficient, which practically coincide with the theoretical values. Non-Gaussian quadrature components can be also used to obtain amplitude and intensity speckles.

**Figure 17:**
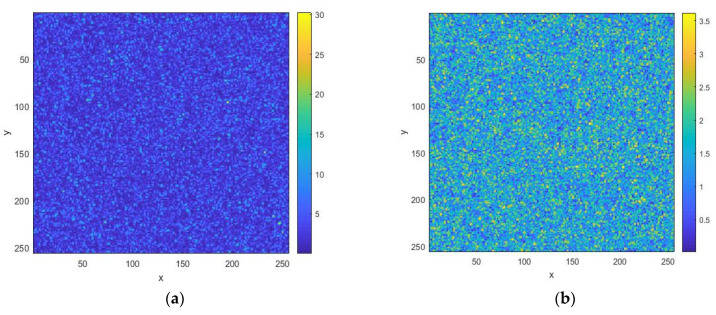
Normalized speckle images for the GG distribution of quadrature components for different shape parameters: (**a**) γ = 1; (**b**) γ = 5. The distances normalized to the radiation wavelength are plotted along the axes *x* and *y*.

The behavior of the amplitude speckle under non-Gaussian distributions of quadrature components is of interest. In Figure 17, the amplitude speckles for the generalized normal distribution of quadrature components are shown.

**Figure 18:**
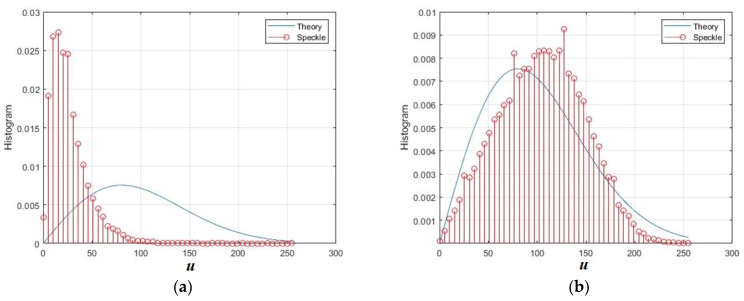
Normalized histograms for amplitude speckle for different shape parameters: (**a**) γ = 1; (**b**) γ = 5.

The histograms obtained are presented in Figure 18 for γ = 1 and γ = 5, where, for comparison, the theoretical densities of chi distributions are shown, which correspond to Gaussian components. This model allows us to obtain a wide variety of values of cumulative coefficients: for γ = 1, we obtain sample values G3 = 1.5 and G4 = 3.78 with KV = 0.72 and CR = 1.66; for γ = 5, the kurtosis is negative, and these values are G3 = 0.018 and G4 = −0.53 with KV = 0.41 and CR = −1683. In the case of Gaussian components, the amplitude speckle had a chi distribution (*L* = 1) with a fixed set of SKE = 0.63; KUR = 0.245; KV = 0.523; and CR = 0.615.

To obtain random variables with the required distribution, the application of a simple nonlinear transformation for each quadrature *x* was investigated, taking into account the transformation to a uniform distribution $z=\Phi^{-1}(x)-0.5$, where Φ(*x*) is the Gaussian distribution function:

$$y=\mu +\beta \cdot sign(z)\cdot {\left[-\ln(1-2abs(z))\right]}^{1/\gamma }.$$

**Figure 19:**
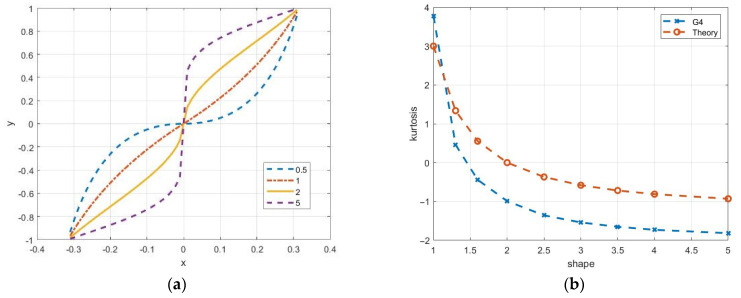
Nonlinear transforming functions to obtain non-Gaussian random variables (**a**) and sampled kurtosis dependency on the shape parameter (**b**).

It is performed significantly faster than the procedure based on the inversion method. The nonlinear function involved in this transformation is shown in Figure 19a for different values of the shape parameter. It is noticeable that with large values of the shape parameter, the steepness increases in the vicinity of the zero values of the argument, which causes a dip in the histograms due to the small number of samples in this range. Thus, the histograms may differ significantly from the theoretical densities. This also affects the characteristics of the generated speckle images. In particular, the sample kurtosis coefficient G4 turns out to be significantly underestimated compared to the theoretical values (Figure 19b).

**Figure 20:**
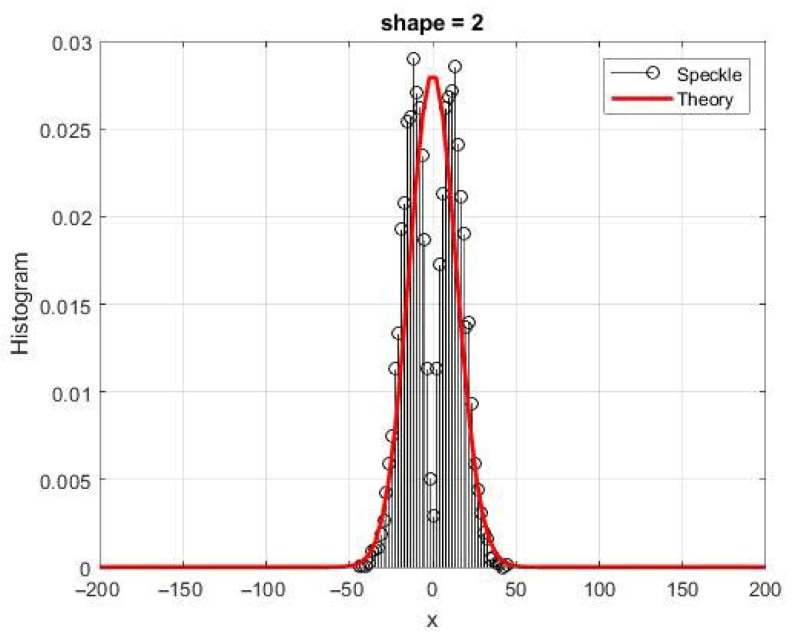
Histogram for the quadrature component for γ = 2 in comparison with a theoretical Gaussian curve.

A nonlinear function in a symmetrical form makes it possible to control the kurtosis of quadrature components in order to obtain, in particular, negative values of this parameter. It also opens up the possibility of a different type of transformation for the real and imaginary components of the speckle, i.e., using an asymmetric transformation to control the skewness of the speckle distribution [29,34]. Figure 20 shows a histogram for the value of the shape parameter γ = 2, at which the probability density should be normal in comparison with the theoretical Gaussian curve. The results are obtained on a 256 × 256 image. Cases where the values of the shape parameter γ are close to 2 should usually be avoided during modeling by applying a real Gaussian approximation.

**Figure 21:**
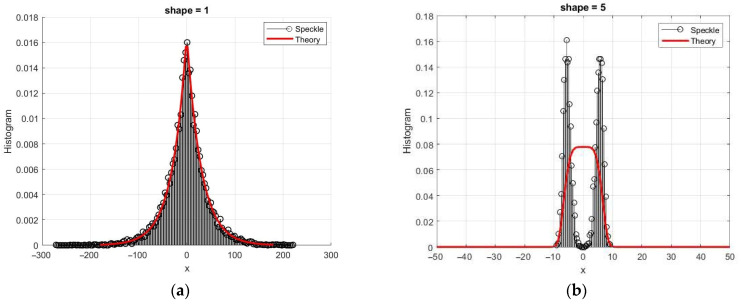
Histograms for the non-Gaussian quadrature component in comparison with theoretical curves: (**a**) γ = 1 (Laplace density); (**b**) γ = 5.

The cases of non-Gaussian distributions for normalized quadrature components are shown in Figure 21.

There are a number of other methods of formation for random variables with a GG distribution [35,36,37], and some of them are based on the inverse incomplete gamma function. The choice of one method or another is related to the convenience of simulation. Methods for the simulation of complex GG distributions are investigated in [38].

## 6. Analyses of Real Radar Images

To illustrate the applicability of the speckle models under consideration, several fragments of real radar images were selected. The images were obtained while the carrier was moving at a speed of 50 km/h at an altitude of 30 m in the stripmap mode at a looking angle of 60°. A pulse mode of radiation was used at a wavelength of 0.1 m with a pulse duration of 2 ns. The images were synthesized over a single look of 2 s. These fragments were analyzed to obtain statistical characteristics and histograms. The characteristics of the various fragments in real radar images differ quite significantly. Although sample estimates of the density parameters provide a qualitative picture of these differences, the various fragments also have common properties characteristic of the parametric models considered.

**Figure 22:**
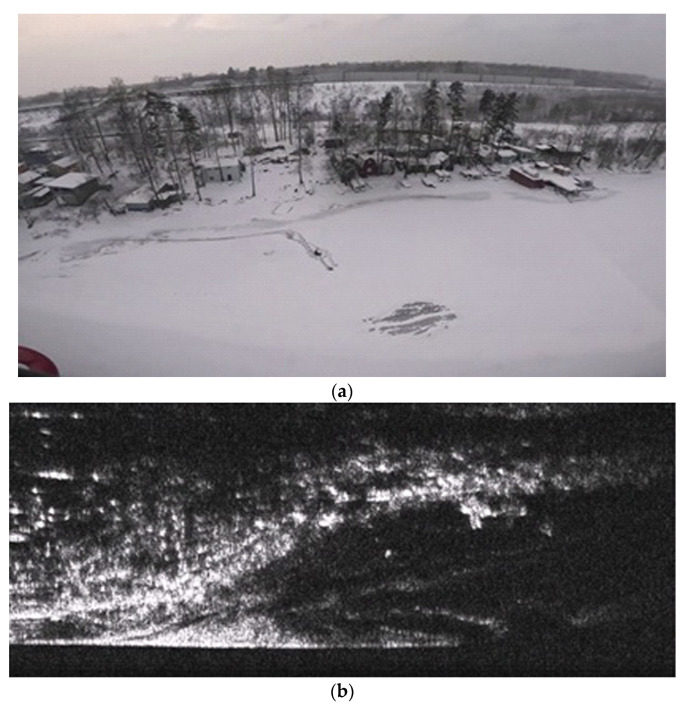
A fragment of the surface captured by a video camera (**a**) and a synthesized speckle image obtained by radar with SAR (**b**).

One of the fragments of the reflected surface is presented in Figure 22. This fragment of a winter scene includes a group of trees, buildings, an icy surface, and the shoreline of a river. The synthesized image clearly highlights buildings and various heterogeneities, which are often objects of interest. It is noticeable that the edge of the coastline differs from that in the video picture because there it is hidden by the icy surface.

**Figure 23:**
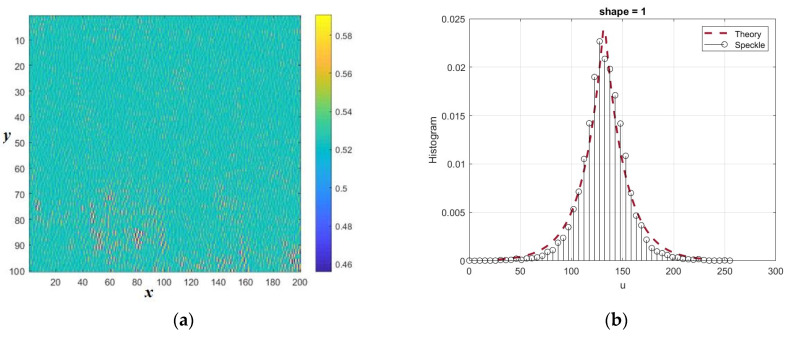
Normalized speckle pattern of one of the quadrature components (**a**) and its histogram (**b**) in comparison with the theoretic density of Laplace (**b**). The distances normalized to the radiation wavelength are plotted along the axes *x* and *y*.

The results of the investigation of a limited section of the forest area are shown in Figure 23a, which shows a 100 × 200 normalized speckle image for one of the quadrature components of the synthesized signal. The corresponding histogram is shown in Figure 23b in comparison with the theoretical density of the Laplace distribution (shape = 1). The sample kurtosis coefficient for this fragment turned out to be more than 5.

**Figure 24:**
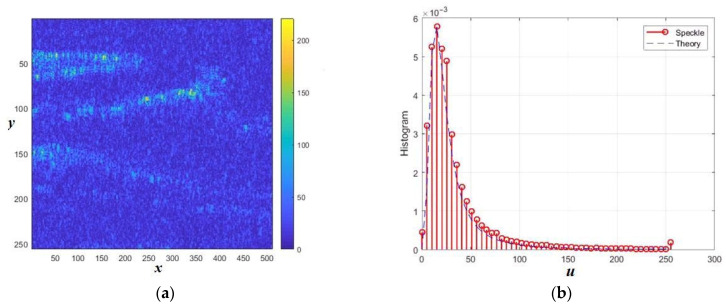
Amplitude speckle pattern (**a**) and its histogram in comparison with the theoretic density in G^0^_A_ class of distributions with parameters α = −1 and *L* = 2 (**b**). The distances normalized to the radiation wavelength are plotted along the axes x and y.

The normalized amplitude speckle pattern in Figure 24a has sample cumulative characteristics G3 = 3.9; G4 = 41.3; and KV = 1.05. The cumulative ratio is CR = 1.7. These parameters are in good agreement with the models in class G^0^_A_. Figure 24b presents the corresponding histogram. The closest theoretical density has parameters α = −1 and *L* = 2.

**Figure 25:**
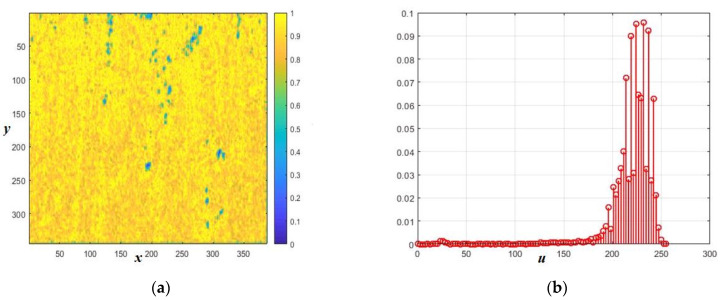
Amplitude speckle pattern with negative skewness (**a**) and its histogram (**b**). The distances normalized to the radiation wavelength are plotted along the axes x and y.

Another fragment of the normalized amplitude speckle image is shown in Figure 25. It has a negative value of the sample skewness coefficient G3 = −4.4 and a rather large kurtosis G4 = 38 with a small coefficient of variation KV = 0.23. Therefore, a model using the generalized Gaussian distribution for complex components is better suited here.

## 7. Discussion

Currently, there are a variety of parametric models for describing speckle images, but there is no clear understanding of the degree of applicability of a particular model, as well as their inherent limitations. The main question is to establish a relationship between the physical parameters of rough surfaces and the parameters of statistical models, or how to select these parameters to obtain the necessary characteristics of speckle images. It requires taking into account not only the statistical characteristics of one-dimensional speckle image distributions but also the analysis of their correlation properties. In this paper, a step has been taken in analyzing the applicability of a mathematical model to describe real fragments of the underlying surface for radar with SAR, which will help solve the main issue.

Based on cumulative analysis, the possibilities and limitations of using practically important speckle models for describing and modeling real fragments of synthesized radar images are investigated, and new comparative results are obtained. For an on-board observation system, along with the reflectivity of the surface, it is important to take into account the geometric sensing scheme, the parameters of the emitted signal, and the synthesis method in the speckle pattern model.

Classical models based on gamma reflection distributions have a physical basis in optics, but they are poorly suited for describing synthesized radar images, since they provide relatively small values of the coefficient of variation, skewness, and kurtosis of the corresponding distributions. The complication due to the introduction of different densities of fluctuations of the reflecting surface and the use of a multiplicative model in some cases leads to constructive results. These include models with amplitude and intensity distributions in classes K-, G-, and G^0^. However, for radar observations, there are variants of speckle patterns characterized by negative skewness and high kurtosis with a low coefficient of variation. The reason for the limitations of known models often lies in the use of the Gaussian distribution of quadrature components.

Non-Gaussian distributions of the complex components of the synthesized speckle pattern are more suitable for such fragments. In particular, the use of a class of generalized Gaussian distributions shows good agreement with experimental characteristics, and some new research results of such models are presented in the article. The transition to non-Gaussian statistics significantly expands the possibilities of modeling but entails some loss of control over the exact distributions of speckle amplitudes and intensities, as well as over their correlation properties. Non-Gaussian complex components can be used to develop a multiplicative model that takes into account fluctuations of the reflecting surface in order to create a constructive and economical parametric model.

The development of such parametric models is important for solving the problems of effective recognition by radar methods of various types of underlying surfaces and the identification of objects of interest on them, including radar video frames generated in videoSAR mode, based on the processing of multi-angle video frames and their interframe, statistical, cluster [39,40], and intelligent analyses. Therefore, further development of these studies involves the definition of datasets and the development of classification methods based on machine learning and neural network technologies.

Classifying underlying surfaces presents a significant challenge, as it does not always allow for the rapid, reliable, and complete determination of the observed surface type. Recent advances demonstrate that, given the availability of large datasets for machine learning, which underpins the neural network, they can be successfully applied to classifying underlying surfaces based on the statistical parameters of reflected radar signals. Thus, the availability of sufficient datasets—the statistical parameters of reflected signals identified in this study—will enable automated classification of underlying surfaces using machine learning methods and neural network technologies.

To implement a neural network classifier for underlying surfaces, it is necessary to determine a set of statistical parameters based on the generated multi-angle video frames for classifying the underlying surfaces and a list of classes for various zones of the observed surfaces. The set of digital radar visual information used to determine the statistical parameter sets includes high-quality and high-resolution video frames. These statistical parameter sets must be determined based on dynamically acquired multi-angle video frames. Such a set of parameters can be the sets of statistical parameters described in this paper. This will provide a representation of various zones of the underlying surfaces, including urban areas, agricultural land, water zones, and others. This dataset allows for the consideration of a wide range of scenarios and conditions, making it an automated tool for the intelligent analysis of big data collected about the earth’s surface.

After defining the set of statistical parameters and their requirements as input for the neural network classifier, the next step is to prepare and split this set of statistical parameters into two separate sets for training and testing the neural network. This separation ensures the independence of the test set, which is important for an objective assessment of the neural network model’s performance. The training set is used for training the neural network model, while the test set is used to test its ability to generalize to data and evaluate its performance on new sets of statistical parameters not used during training. This helps prevent overfitting and ensures that the neural network model is capable of working with new data, i.e., data not used during training.

To implement training, it is first necessary to analyze neural network architectures and select a neural network suitable for the task at hand. Next, one must determine the structure of the selected neural network, taking into account the specifics of the input data: statistical parameters determined from radar video frames. Then, one must determine the requirements for the video frames used to train the neural network. Next is preparing a set of statistical parameters for training the neural network. The process of preparing training data is crucial, as it will determine the subsequent quality of its performance. Finally, it is necessary to train the neural network using the prepared data.

After training the neural network on the data, it is tested on data not used in the training to verify its performance and assess its completeness and accuracy. If the neural network fails to achieve the required accuracy and completeness for classifying the Earth’s surface zones after testing, it should be retrained. This will improve the neural network’s functionality. The process of adjusting the network’s weights and parameters during retraining is repeated until acceptable accuracy and completeness are achieved.

The implementation of the neural network classifier in the manner described above will allow for the rapid determination of surface types based on the statistical parameters of video frame zones to assess dynamically changing radar conditions that are independent of meteorological or lighting conditions.

## 8. Conclusions

A set of statistical models is presented and analyzed to provide flexible and adequate descriptions of intensity, amplitude, and quadrature speckle components for SAR observations of the underlying surface. The simulation results are in good agreement with the characteristics of real speckle images for several representative terrain fragments.

These results can serve as a basis for constructing reference sets of radar characteristics of reflected signals. Such reference sets may support the analysis of variations in reflection geometry during flight and in multi-radar video SAR group operation.

## Data Availability

All data supporting the reported results are public.

## Abbreviations

The following abbreviations are used in this manuscript:

SAR	Synthetic Aperture Radar
GG	Generalized Gaussian (distribution)
FFT	Fast Fourier Transform
KV	Coefficient of Variation
SKE	Coefficient of Skewness
KUR	Coefficient of Kurtosis
CR	Cumulative Ratio
G3	Sample Skewness
G4	Sample Kurtosis
